# Cardiovascular Disease and Breast Cancer Stage at Diagnosis

**DOI:** 10.1001/jamanetworkopen.2024.52890

**Published:** 2025-01-02

**Authors:** Ivan Angelov, Allen M. Haas, Elizabeth Brock, Lingfeng Luo, Jing Zhao, Benjamin D. Smith, Sharon H. Giordano, Nicholas J. Leeper, Kevin T. Nead

**Affiliations:** 1 Department of Epidemiology, University of Texas MD Anderson Cancer Center, Houston; 2 School of Medicine, Baylor College of Medicine, Houston, Texas; 3 Department of Health Services Research, University of Texas MD Anderson Cancer Center, Houston; 4 Division of Vascular Surgery, Department of Surgery, Stanford University School of Medicine, Stanford, California; 5 Stanford Cardiovascular Institute, Stanford, California; 6 Department of Breast Medical Oncology, University of Texas MD Anderson Cancer Center, Houston; 7 Division of Cardiovascular Medicine, Department of Medicine, Stanford University School of Medicine, Stanford, California; 8 Department of Radiation Oncology, University of Texas MD Anderson Cancer Center, Houston

## Abstract

**Importance:**

Cardiovascular disease (CVD) and cancer are the leading causes of mortality in the US. Large-scale population-based and mechanistic studies support a direct effect of CVD on accelerated tumor growth and spread, specifically in breast cancer.

**Objective:**

To assess whether individuals presenting with advanced breast cancers are more likely to have prevalent CVD compared with those with early-stage breast cancers at the time of diagnosis.

**Design, Setting, and Participants:**

This population-based case-control study used data from the Surveillance, Epidemiology, and End Results–Medicare linked databases from 2009 to 2020. The analysis was completed from May 2023 to August 2024. Participants were female patients aged at least 66 years diagnosed with invasive breast cancer. Cases were matched with controls by breast cancer stage at diagnosis and propensity scores using factors known to be associated with delayed cancer diagnosis.

**Exposure:**

Prevalent CVD prior to breast cancer diagnosis.

**Main Outcomes and Measures:**

The outcome of interest was the odds of locally advanced (T3-4 or N+) or metastatic (M+) breast cancer status at diagnosis.

**Results:**

The full analytic cohort included 19 292 matched individuals, with median (IQR) age 73 (70-79) years, of whom 1676 (8.7%) were Black and 16 681 (86.5%) were White; 9478 individuals (49.1%) had prevalent CVD. Propensity score–matched, multivariable-adjusted models found that individuals with locally advanced or metastatic breast cancer at diagnosis had statistically significantly increased odds of prevalent CVD (odds ratio [OR], 1.10; 95% CI, 1.03-1.17; *P* = .007). This association was observed among hormone receptor–positive (OR, 1.11; 95% CI, 1.03-1.19; *P* = .006) but not hormone receptor–negative (OR, 1.02; 95% CI, 0.86-1.21; *P* = .83) breast cancer. ORs were directionally consistent when separately examining locally advanced (OR, 1.09; 95% CI, 1.02-1.17; *P* = .02) and metastatic (OR, 1.20; 95% CI, 0.94-1.54; *P* = .15) disease, among all receptor subtypes.

**Conclusions and Relevance:**

This case-control study found that individuals with more advanced breast cancer at diagnosis were more likely to have prevalent CVD. This finding may be specific to hormone receptor–positive and *ERBB2*-negative (formerly *HER2*) disease. Future studies are needed to confirm these findings and investigate interventions to improve patient outcomes, including personalized cancer screening.

## Key Points

**Question:**

Are individuals with advanced breast cancer at diagnosis more likely to have prevalent cardiovascular disease?

**Findings:**

This case-control study of 19 292 individuals found that patients with more advanced breast cancer at diagnosis had a statistically significant 10% increased odds of prevalent cardiovascular disease, even when accounting for factors associated with delayed cancer diagnosis.

**Meaning:**

These findings suggest that individuals with cardiovascular disease may be at an increased risk of an advanced breast cancer at diagnosis, which could inform personalized screening recommendations.

## Teaser

This case-control study examines whether individuals presenting with more advanced breast cancers at diagnosis were more likely to have prevalent cardiovascular disease compared with individuals with early-stage breast cancers at diagnosis.

## Introduction

Cardiovascular disease (CVD) and cancer are the leading causes of mortality in the US and share well-established risk factors.^1^ However, emerging evidence suggests that CVD may play a direct causal role in the etiology and progression of cancer. Indeed, we have used large population-level studies to show an association between CVD and incident cancer risk, independent of traditional risk factors.^2,3^ Building on this evidence, multiple mechanistic investigations have revealed that tumor growth is accelerated in the context of heart failure, cardiac remodeling, and myocardial infarction (MI), suggesting a possible direct causal link.^4,5,6^ This effect is notable in breast cancer, where CVD induces an immunosuppressive state fostering accelerated breast tumor cell growth and spread.^7^ We therefore hypothesized that individuals with prevalent CVD might present with more advanced breast cancer at diagnosis.

## Methods

This case-control study was exempted from review and informed consent by the University of Texas MD Anderson Cancer Center institutional review board because patient data were deidentified and publicly available. This study followed the Strengthening the Reporting of Observational Studies in Epidemiology (STROBE) reporting guideline.

### Cohort Selection

In this case-control study, we used the Surveillance, Epidemiology, and End Results–Medicare linked databases. We identified female patients aged at least 66 years diagnosed with invasive breast cancer from 2010 to 2019 (eTable 1 in Supplement 1). We included individuals with a screening mammogram in the 2 years prior to breast cancer diagnosis to account for confounding according to health care behavior and control for the duration of breast cancer prior to clinical detection.^8^

### Exposures and Covariables

CVD status was determined from the 3 to 24 months prior to breast cancer diagnosis to avoid concurrent diagnoses that may represent incidental findings and to establish a timeline of preexisting CVD prior to breast cancer diagnosis.^2^ Comorbidities were identified in the 24 months prior to breast cancer diagnosis. Derivation of variables are defined in eTable 2 in Supplement 1. Race and ethnicity were extracted from Surveillance, Epidemiology, and End Results data abstracted from medical records.^9^ Race was categorized as Black, White, or other (eg, American Indian or Alaska Native or Asian or Pacific Islander). Ethnicity was classified as Hispanic or non-Hispanic. Race and ethnicity were included as potential confounding factors. Variable values with unknown or missing data (race, marital status, region) were considered as a separate category or, where necessary to facilitate matching, grouped with other.

### Statistical Analysis

We used a case-control design based on cancer stage at diagnosis to examine the odds of prevalent CVD. Our primary analysis compared CVD status among individuals with early-stage breast cancer (T1-T2 and N0 and M0) vs those with advanced disease (T3-T4 or N+ or M+). We a priori stratified our analysis by receptor subtype and examined patients with locally advanced (T3-4 or N+ and M0) and metastatic (M+) disease, separately.

We conducted a greedy nearest neighbor propensity score 1:1 caliper (0.25)^10^ exact match of individuals with advanced disease (locally advanced or metastatic) to those with early-stage breast cancer. The propensity score used factors with evidence for delayed cancer diagnosis: age, race and ethnicity, Medicaid eligibility, urban or rural residence, marital status, region, and quartile of number of health care interactions (unique days with an inpatient or outpatient claims code) in the 24 months prior to cancer diagnosis.^11,12^ These factors may also be associated with risk of CVD.^13,14^ To account for our matched study design, we used conditional logistic regression to examine the association between CVD and breast cancer stage at diagnosis further adjusting for comorbid conditions, including chronic obstructive pulmonary disease, diabetes, hypertension, hyperlipidemia, and chronic kidney disease.

Tests were considered statistically significant with 2-sided *P* < .05. Analyses were conducted using SAS Enterprise Guide (SAS Institute) version 7.15 and R version 4.3.2 (R Project for Statistical Computing).

## Results

Our full analytic cohort included 19 292 individuals (median [IQR] age, 73 [70-79] years) with breast cancer. The cohort included 1676 Black individuals (8.7%), 16 681 White individuals (86.5%), and 935 individuals (4.8%) who had other or unknown race; 1058 individuals (5.5%) were Hispanic and 18 234 individuals (94.5%) were non-Hispanic. In our matched cohort, 9478 individuals (49.1%) of individuals had CVD, with 8675 individuals (91.5%) having had their disease detected in the 13 to 24 months prior to breast cancer diagnosis. There were 9646 individuals with early-stage breast cancer matched with 9646 individuals with locally advanced or metastatic disease. Demographic and clinical data for the matched groups are presented in the Table.

**Table.** Demographic Data for the Analytic Cohort Stratified by Disease Stage at Diagnosis

Characteristic	Patients, No. (%) (N = 19 292)		*P* value
	Early stage (n = 9646)	Locally advanced or metastatic (n = 9646)	
Stage grouping			
Early stage	9646 (100)	NA	NA
Locally advanced	NA	8956 (92.9)	
Metastatic	NA	690 (7.1)	
Age, y			
66-70	2964 (30.7)	2938 (30.5)	.81
71-75	2995 (31.0)	2988 (31.0)	
76-80	2022 (21.0)	2005 (20.8)	
81-85	1105 (11.5)	1115 (11.6)	
≥86	560 (5.8)	600 (6.2)	
Race			
Black	831 (8.6)	845 (8.8)	.07
White	8381 (86.9)	8300 (86.0)	
Other or unknown^a^	434 (4.5)	501 (5.2)	
Ethnicity			
Hispanic	524 (5.4)	534 (5.5)	.75
Non-Hispanic	9122 (94.6)	9112 (94.5)	
Dual Eligibility			
No	8394 (87.0)	8365 (86.7)	.54
Yes	1252 (13.0)	1281 (13.3)	
Rural			
Urban	8110 (84.1)	8079 (83.8)	.54
Rural	1536 (15.9)	1567 (16.2)	
Marital Status			
Married	4771 (49.5)	4726 (49.0)	.90
Separated or divorced	906 (9.4)	922 (9.6)	
Single	754 (7.8)	771 (8.0)	
Widowed	2764 (28.7)	2754 (28.6)	
Other or unknown	451 (4.7)	473 (4.9)	
Region			
Northeast	1560 (16.2)	1577 (16.3)	.93
Midwest	NR^b^	NR^b^	
Southeast	2540 (26.3)	2544 (26.4)	
West	4428 (45.9)	4415 (45.8)	
Unknown	NR^b^	NR^b^	
Visits			
1-15	2368 (24.5)	2366 (24.5)	.98
16-27	2375 (24.6)	2392 (24.8)	
28-45	2430 (25.2)	2409 (25.0)	
≥46	2473 (25.6)	2479 (25.7)	
CKD	701 (7.3)	763 (7.9)	.09
COPD	1376 (14.3)	1435 (14.9)	.23
Hyperlipidemia			
No	2259 (23.4)	2414 (25.0)	.009
Yes	7387 (76.6)	7232 (75.0)	
Hypertension	7146 (74.1)	7333 (76.0)	.002
Diabetes	2783 (28.9)	3077 (31.9)	<.001
Cancer type			
HR+/*ERBB2*+	910 (9.4)	910 (9.4)	>.99
HR−/*ERBB2*+	393 (4.1)	393 (4.1)	
HR+/*ERBB2*−	7292 (75.6)	7292 (75.6)	
HR−/*ERBB2*−	1051 (10.9)	1051 (10.9)	
CVD	4636 (48.1)	4842 (50.2)	.003

Abbreviations: −, negative; +, positive; CKD, chronic kidney disease; COPD, chronic obstructive pulmonary disease; CVD, cardiovascular disease; HR, hormone receptor; NA not applicable.

^a^ Includes American Indian or Alaska Native and Asian or Pacific Islander.

^b^ In accordance with the Surveillance, Epidemiology, and End Results–Medicare data use agreement, cells are suppressed to avoid identification of cell sizes of fewer than 11 patients.

**Figure 1.**
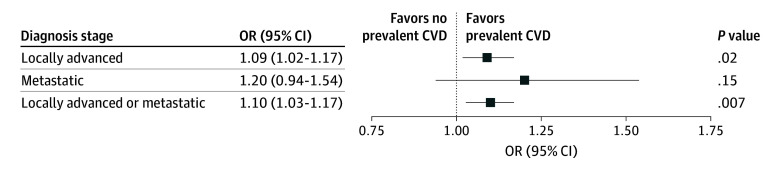
Association of Prevalent Cardiovascular Disease (CVD) With Breast Cancer Stage at Initial Diagnosis For all comparisons, the reference group is early-stage disease at diagnosis (T1-T2 and N0 and M0). OR indicates odds ratio.

In our primary analysis (Figure 1), we found that individuals with locally advanced or metastatic breast cancer at diagnosis had statistically significantly increased odds of prevalent CVD (odds ratio [OR], 1.10; 95% CI, 1.03-1.17; *P* = .007). We observed a similar finding when examining locally advanced (T3-T4 or N+) disease (OR, 1.09; 95% CI, 1.02-1.17; *P* = .02). When examining metastatic disease at diagnosis, our findings were directionally consistent but not statistically significant (OR, 1.20; 95% CI, 0.94-1.54; *P* = .15).

**Figure 2.**
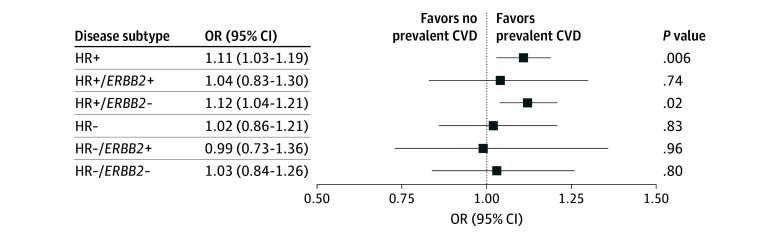
Association of Prevalent Cardiovascular Disease (CVD) With Advanced Breast Cancer at Diagnosis According to Receptor Subtype Advanced breast cancer was defined as T3 to T4, N+, or M+. For all comparisons, the reference group is early-stage disease at diagnosis (T1-T2 and N0 and M0). − indicates negative; +, positive; HR, hormone receptor; OR, odds ratio.

Stratification of our primary analysis by receptor subtype demonstrated that the observed association was present in hormone receptor–positive (OR, 1.11; 95% CI, 1.03-1.19; *P* = .006) but not hormone receptor–negative (OR, 1.02; 95% CI, 0.86-1.21; *P* = .83) breast cancer (Figure 2). This finding was primarily driven by the strength of the association in hormone receptor–positive and *ERBB2*-negative (formerly *HER2*) disease.

## Discussion

This large case-control study found that patients with CVD were more likely to present with locally advanced or metastatic breast cancer at diagnosis. Importantly, our analyses were conducted among patients who recently underwent breast cancer screening, and we accounted for factors associated with delayed cancer diagnosis. These results are highly consistent with mechanistic data supporting a causal effect of CVD on breast cancer growth and metastasis and build on an evolving body of evidence supporting a causal link between CVD and cancer. Future studies are needed to determine whether individuals with CVD may benefit from personalized breast cancer screening approaches, which may be particularly relevant in the setting of current conflicting screening recommendations regarding age and frequency.^8^

Prior studies support our findings. The Atherosclerosis Risk In Communities study demonstrated that adherence to cardiovascular health metrics was associated with a 50% decreased risk of incident cancer, particularly for breast, lung, and colorectal cancers.^15^ Initiation of statin therapy following breast cancer diagnosis is associated with improved breast cancer–specific survival.^16^ A hospital-based cohort study, including 32 095 individuals with CVD, found a 2-fold increased risk of cancer among patients with CVD.^3^ Additionally, retrospective cohort studies support an association between heart failure and increased cancer incidence.^4,5^

Building on these results, our group previously examined more than 27 million individuals and found a 13% increased risk of cancer among individuals with CVD, independent of traditional shared risk factors, including smoking.^2^ In this prior analysis, we observed a decreased risk of breast cancer among individuals with atherosclerotic CVD compared with those without CVD (hazard ratio, 0.86; 95% CI, 0.76-0.97).^2^ However, compared with the current analysis, this prior study^2^ examined incidence rather than stage at diagnosis, relied on insurance claims rather than cancer registry data, did not account for breast cancer–specific factors (eg, stage, receptors, prior mammograms), and examined a significantly younger cohort (mean age, 43 years) in whom the breast cancer subtype with the strongest association in the current analysis (hormone receptor positive and *ERBB2* negative) is least common.^17^ Additionally, the protective association of CVD with breast cancer risk previously observed was not statistically significant with adjustment for multiple testing. Therefore, further studies are needed to understand the impact of CVD on breast cancer incidence and whether this association differs from potential effects on stage at diagnosis.

Murine models provide strong support for a causal link between CVD and cancer. Heart failure following MI in mouse models prone to developing intestinal tumors results in enhanced tumor growth.^5^ Models of early hypertrophic cardiac remodeling additionally support promotion of tumor growth and metastases via direct cardiovascular and cancer crosstalk.^6^ Relevant to the current analysis, a study by Koelwyn et al^7^ using a breast cancer mouse model of MI observed a 2-fold increase in Ki67-positive tumor cells, increased primary tumor volume, and enhanced metastatic spread compared with sham-treated mice.^7^ Analysis of intratumoral immune cells showed increased tumoral CD45-positive leukocytes in the MI group with further immunophenotyping, consistent with an MI-induced immunosuppressive state allowing accelerated tumor growth. Based on these prior data, we hypothesized, and ultimately found, that prevalent CVD was associated with more advanced breast cancer at diagnosis. A direct impact of CVD on a more aggressive underlying breast cancer biology may at least partially explain the increased breast cancer–specific mortality observed among patients with CVD.^18^

Our results were primarily driven by the observed association among individuals with hormone receptor–positive and *ERBB2*-negative disease. This finding is consistent with existing mechanistic data supporting a role of CVD in accelerated breast tumor cell growth and spread^7^ where the mouse models most commonly develop hormone receptor–positive breast cancer.^19,20^ Additionally, existing data supporting direct crosstalk between CVD and cancer specifically indicate a potential effect of acute cardiovascular insults.^4,5,6,7^ If acute events drive the biological link between CVD and cancer, then more indolent cancer types, such as hormone receptor–positive and *ERBB2*-negative breast cancer, may have a larger time window to be effected prior to initial diagnosis. However, our subtype analysis should be interpreted with caution, given insufficient power to show a statistically significant differential association across breast cancer subtypes.

### Limitations

This study has some limitations. Our study was observational, does not demonstrate causality, and is susceptible to residual bias and confounding. Testing of our hypothesis in a prospective design is needed to better investigate the observed association between CVD and breast cancer stage at diagnosis. There is potential for misclassification of CVD using diagnoses and procedure codes. We are unable to control for some potential confounding factors, including smoking. Our analytic cohort was primarily White patients, which may impact generalizability of our findings. Additionally, some of our analyses, such as by subtype, had low power. As we used a case-control study design, we are unable to provide information regarding the absolute impact of CVD on breast cancer stage at diagnosis in the unselected population. We did not have sufficient data regarding prior hormone replacement therapy use, which may be a confounding factor, given potential associations with both CVD and breast cancer.

## Conclusions

This retrospective case-control study found an association between prevalent CVD and more advanced stage of breast cancer at diagnosis, independent of factors associated with delayed diagnosis and shared risk. Further studies are needed to determine whether individuals with CVD would benefit from personalized screening approaches.

## Notes

Supplement 1. **eTable 1. **Analytic Cohort Selection **eTable 2. **Approach to Identify Exposures, Outcomes, and Covariables

Supplement 2.
Data Sharing Statement
