# Error in Table

**DOI:** 10.1001/jamanetworkopen.2025.22553

**Published:** 2025-06-24

In the Original Investigation titled “Cardiovascular Disease and Breast Cancer Stage at Diagnosis,”^1^ published January 2, 2025, there were errors in the presentation of suppressed data in the Table. These should have appeared as not reported. This article has been corrected.^1^
