# Supplementary material for: Cardiovascular Disease and Breast Cancer Stage at Diagnosis
Source: JAMA Netw Open. 2025 Jan 2;8(1):e2452890. doi: 10.1001/jamanetworkopen.2024.52890 (PMC11696447; doi:10.1001/jamanetworkopen.2024.52890)
Supplement: Supplement 1. — eTable 1. Analytic Cohort Selection eTable 2. Approach to Identify Exposures, Outcomes, and Covariables [file jamanetwopen-e2452890-s001.pdf]

# Supplemental Online Content

Angelov I, Haas AM, Brock E, et al. Cardiovascular disease and breast cancer stage at diagnosis. *JAMA Netw Open*. 2025;8(1):e2452890. doi:10.1001/jamanetworkopen.2024.52890

**eTable 1.** Analytic Cohort Selection
**eTable 2.** Approach to Identify Exposures, Outcomes, and Covariables

This supplemental material has been provided by the authors to give readers additional information about their work.

© 2025 Angelov I et al. *JAMA Network Open.*

**eTable 1:** Analytic cohort selection.

| Criteria | n |
|---|---|
| 1. Select patients with a primary breast cancer diagnosed between 2010 and 2019. | 539,958 |
| 2. Select patients who are female. | 535,756 |
| 3. Select patients who are age 66 years and older. | 316,026 |
| 4. Select patients with continuous part A and B coverage and no HMO coverage for the 24-months prior to diagnosis. | 173,327 |
| 5. Select patients with complete staging data and without *in situ* disease. | 85,644 |
| 6. Select patients with complete hormone receptor status information. | 81,043 |
| 7. Remove patients with unclear CVD status. * | 70,117 |
| 8. Remove patients who did not receive a screening mammogram in the two years before breast cancer diagnosis. | 46,984 |
| 9. Propensity score matched cohort on breast cancer stage at diagnosis. | 19,292 |

CVD, cardiovascular disease; HMO, health maintenance organization; n, number
* Individuals with only one outpatient CVD code were excluded due to unclear CVD status.

© 2025 Angelov I et al. *JAMA Network Open.*

**eTable 2**: Approach to identify exposures, outcomes, and covariables.

| **Variables** | **Codes/Algorithms** |
|---|---|
| Breast Cancer | C501 – C509 (ICD-O-3) |
| Screening mammogram | Screening mammogram defined as a screening mammogram specific CPT code OR any mammogram CPT code with GG modifier per: Richman IB, Gross CP. Estimating Breast Cancer Overdiagnosis After Screening Mammography Among Older Women in the United States. Ann Intern Med. Mar 2024;177(3):403-404. doi:10.7326/L23-0485 |
| Screening Mammogram | 76092, 77057, 77067, G0202, G0203, 76083, 76085, 77052, 77063 |
| Other Mammogram Codes (used if present with modifier GG) | 76091, 77056, 77066, G0204, G0205, 76090, 77055, 77065, G0206, G0207, 76082, 77051, 77061, 77062, G0279, G0236 |
| Chronic obstructive pulmonary disease (derived from CMS Chronic Conditions Data Warehouse) | ICD9: 490, 496, 491.0, 491.1, 491.8, 491.9, 492.0, 492.8, 494.0, 494.1, 491.20, 491.21, 491.22<br>ICD10: J40, J42, J41.0, J41.1, J41.8, J43.0, J43.1, J43.2, J43.8, J43.9, J44.0, J44.1, J44.9, J47.0, J47.1, J47.9 |
| Cardiovascular disease, hyperlipidemia, hypertension, chronic kidney disease, diabetes | Defined per: Bell CF, Lei X, Haas A, et al. Risk of Cancer After Diagnosis of Cardiovascular Disease. JACC CardioOncol. Aug 2023;5(4):431-440. doi:10.1016/j.jaccao.2023.01.010<br>• Cardiovascular disease status was determined from the 24-months prior to cancer diagnosis using 1) 2 or more separate International Classification of Disease (ICD), 9th (ICD-9) or 10th (ICD-10) codes from an outpatient setting at least 30 days apart, 2) one inpatient code, or 3) one procedure code.<br>    o Individuals with only one outpatient cardiovascular disease code were excluded due to unclear CVD status.<br>• Comorbidities were identified by the presence of any relevant code in the two years prior to diagnosis |

© 2025 Angelov I et al. *JAMA Network Open.*
