# Supplementary material for: Cardiovascular Disease and Breast Cancer Stage at Diagnosis
Source: JAMA Netw Open. 2025 Jan 2;8(1):e2452890. doi: 10.1001/jamanetworkopen.2024.52890 (PMC11696447; doi:10.1001/jamanetworkopen.2024.52890)
Supplement: Supplement 2. — Data Sharing Statement [file jamanetwopen-e2452890-s002.pdf]

Data Sharing Statement

Angelov. Cardiovascular Disease and Breast Cancer Stage at Diagnosis. *JAMA Netw Open*. Published January 02, 2025. doi:10.1001/jamanetworkopen.2024.52890

Data
Data available: No

Additional Information
Explanation for why data not available: We are not permitted to share SEER-Medicare data per the data use agreement.
